# Combatting a "Twin-demic": A quantitative assessment of COVID-19 and influenza vaccine hesitancy in primary care patients

**DOI:** 10.34172/hpp.2021.22

**Published:** 2021-05-19

**Authors:** Kemmian D. Johnson, Oluwatomi Akingbola, Jessica Anderson, Jennifer Hart, Andrew Chapple, Che'la Woods, Karen Yeary, Angela McLean

**Affiliations:** ^1^Department of Internal Medicine, Louisiana State University Health Sciences Center, 1542 Tulane Ave Suite 436 New Orleans, LA 70112, USA; ^2^School of Medicine, Tulane University, 1430 Tulane Ave, New Orleans, LA 70112; ^3^School of Medicine, Louisiana State University Health Sciences Center, 1542 Tulane Ave Suite 436 New Orleans, LA 70112, USA; ^4^School of Public Health, Biostatistics, Louisiana State University Health Sciences Center, 1542 Tulane Ave Suite 436 New Orleans, LA 70112, USA; ^5^Rosalind Franklin University of Medicine and Science- Chicago Medical School, 3333 Green Bay Rd, North Chicago, IL 60064, USA; ^6^Roswell Park Comprehensive Cancer Center, Elm and Carlton Streets Buffalo, NY 14263

**Keywords:** COVID-19, 2019 Novel coronavirus vaccine, COVID-19 vaccines, Influenza vaccine, Public health

## Abstract

**Background:** Public health officials anticipate severe health outcomes amidst the circulation of two major viruses, severe acute respiratory syndrome coronavirus 2 (SARS-CoV-2) and influenza. This study investigated intent to be vaccinated against COVID-19 and influenza, and sought to identify attitudes towards vaccines and barriers for vaccine acceptance.

**Methods:** This observational cross-sectional study was conducted in the Louisiana State University Medicine Clinic from September 2020 to December 2020. Intent to be vaccinated against the COVID-19 and influenza virus was assessed through a brief questionnaire. Additionally, hesitancy and attitudes regarding vaccines were ascertained using validated 5-point Likert scales. In total, 280 patients completed the questionnaire.

**Results:** A total of 248 patients were included in the final analysis. Overall 167 (67%, 95% CI = 61.1-73.0%) of patients were unsure or did not intend to be vaccinated against COVID-19, while only 19.3% (95% CI = 14.4-24.5%) were unsure or did not intend to be vaccinated against the influenza vaccine. Reasons for COVID-19 vaccine hesitancy included concern regarding side effects, fear of getting sick from the vaccine, and the absence of vaccine recommendations from their doctor. Concerningly, African American patients demonstrated decreased likelihood of receiving the COVID-19 vaccine.

**Conclusion:** This survey revealed that only 1 in 3 adults intended to be vaccinated against COVID-19, while 8 out of 10 adults intended to receive the influenza vaccine. Patients who intended on getting the COVID-19 vaccine were less likely to be African American. Given the degree of hesitancy against COVID-19 vaccination, a multifaceted approach to facilitate vaccine uptake that includes vaccine education, behavioral change strategies, and health promotion, is paramount.

## Introduction

COVID-19, the disease caused by the severe acute respiratory syndrome coronavirus 2 (SARS-CoV-2), has spread worldwide and has been declared a pandemic with over 75 million people infected and 1.7 million related deaths.^1^ A number of clinical trials have been launched to investigate potential therapies to treat COVID-19. However, a COVID-19 vaccine is ultimately the most effective long-term method to controlling the pandemic.^2^ Global research efforts have led to rapid development of a new vaccine using messenger RNA (mRNA). Both the Pfizer (n=43 538) and Moderna (n=30 350) COVID-19 vaccine trials reported at least 94-95% vaccine efficacy and have begun distribution.^3,4^

In launching the new COVID-19 vaccines, public health officials also advise of the potential for co-circulation and co-infection with both SARS-CoV-2 and the influenza virus as “flu season” is underway.^5^ As such, influenza vaccine administration remains a critical step to reducing the severity of acute respiratory illnesses and preventing associated morbidity and mortality.

However, the World Health Organization recently reported significant declines in the number of people receiving vaccines globally, which reflects a recent international trend known as “vaccine hesitancy”.^6^ The Strategic Advisory Group of Experts defines vaccine hesitancy as “a delay in acceptance or refusal of vaccination despite availability of vaccination services”.^7^ In the 2018-19 year, less than half of U.S. adults received an annual influenza vaccine, as the overall vaccine coverage remained at about 45%.^8^ Globally, vaccine hesitancy has increased with a concurrent decrease in vaccine rates, and an increase in disease outbreak of vaccine-preventable diseases.^8^

Healthcare workers are at the frontline of the COVID-19 pandemic and a priority target group for COVID-19 vaccines, with a general willingness to receive the vaccines.^9^ However, little data exists regarding the willingness or hesitancy of U.S. patients and/or the general population to receive the new COVID-19 vaccine once available. Further, given the potential for severe illness with coinfection, little data exists regarding patient interest and intention to receive both the flu vaccine and COVID-19 vaccine.

## Materials and Methods

### Study design, participants and procedure 

The study population comprised of adult patients residing in Louisiana, who were patients in the Internal Medicine Clinic. Patients were invited to participate in the study and enrolled in-person during registration for their scheduled clinic appointments. After informed consent was obtained at the time of study enrollment, patients were given the survey to complete. Patients completed and returned surveys to registration staff prior to leaving clinic. Data for the present study were from September 15, 2020 through December 04, 2020.

### Measures

Demographics of gender, age, race/ethnicity, education, and total household income were assessed.

Vaccine Intent: *Intention to receive the COVID-19 vaccine* was assessed by asking, “Once a COVID-19 vaccine is available, do you plan to get the vaccine?” followed by the responses “yes”, “no” or “unsure”. Participants who responded “no” or “unsure” were asked to provide a reason for choosing “no” or “unsure”. Response options were: “I am concerned about side effects of the vaccine”, “Vaccines do not work well/are ineffective”, “Costs/Too expensive”, “I do not have health insurance”, “I do not like needles and shots”, “I do not have the time to get the vaccine”, “My doctor did not recommend the vaccine”, “I believe I am healthy and do not need to get the vaccine”, “I will get sick if I receive the vaccine”, “I have a medical illness that prevents me from getting vaccines” and “Other reason”. *Intention to receive the influenza vaccine* was measured by asking, “Do you plan to get the vaccine this year?” followed by the responses “yes”, “no” or “unsure”. Similar to the intention to receive the COVID-19 vaccine question described above, participants who responded “no” or “unsure” were asked to provide a reason for choosing “no” or “unsure” and were provided with 11 response options (e.g. I am concerned about the side effects of the vaccine).

Vaccine Hesitancy and participants’ attitudes and beliefs regarding vaccines was assessed through a modified version of the validated 24-item “Vaccine Hesitancy” survey, which was originally developed in 2015 by the SAGE group.^7^ Our scale for measuring vaccine hesitancy comprised 8 elements. To increase the scale’s relevance to our study, we modified the wording of the scale to address general, COVID-19 and influenza vaccines as opposed to childhood vaccines. For example, the statement “Getting vaccines is a good way to protect my child from disease” was converted to “Getting the COVID-19 vaccine is a good way to protect myself from disease” and “Getting the influenza vaccine is a good way to protect myself from disease”. Response categories were detailed using a five-point Likert scale (‘strongly disagree’, ‘disagree’, ‘unsure’, ‘agree’ and ‘strongly agree’). The psych package in R statistical software to was used to compute the Cronbach’s alpha for our survey items regarding vaccine intent. The raw alpha score was 0.89 (95% CI 0.87-0.91) indicating that the survey has good reliability.

### Statistical Analysis

R statistical software version 4.0.2 was used for all analyses. Continuous covariates were summarized by mean (SD) and categorical covariates were summarized by count (%). Categorical covariates were compared between the “yes” and “no/unsure” COVID-19 vaccine groups using Fisher exact tests, which implicitly assume that the categorical covariates in each group follow a multinomial distribution.

Normality was assumed for means of continuous covariates which were compared using two sample *t* tests. This assumption is reasonable due to the large sample size and results from the central limit theorem.

Multivariable logistic regression was performed to determine factors that predicted a “yes” COVID-19 vaccine intent. Patient level covariates: Male gender, Black race, Hispanic ethnicity, > High school education, Flu history, Flu vaccine intent, low income, age; were entered as fixed effects in the model using the glm() function in R statistical software. Missing data was assumed to be missing at random and only complete cases were used for the logistic regression, meaning 18 observations were not used for that analysis adjusted odds ratios (aORs) and associated 95% confidence intervals were presented for each fixed effect.

## Results

A total of 280 patients completed the survey; 248 were included in the final analysis. Two patients were removed for missing data, and 30 were removed due to having a previous history or diagnosis of COVID-19.

The majority of participants were African American (65.2%), female (57%) and over 60 years of age (41%). Various participant educational attainment levels were recorded, with a majority (63%) reporting having a high-school diploma or less. Most participants (66%) reported an annual income less than $25 000. Approximately three fourths (70.4%) of participants reported having received the influenza vaccine during the previous year. Other patient characteristics are reported in Table 1.

### Vaccine intent

A total of 33% (n=81) of patients surveyed reported intent to receive the COVID-19 vaccine, while 67% (n=167) reported no intention to receive the vaccine or were unsure whether they would receive the vaccine. On the other hand, a significant majority of respondents (80.7%), reported intention to receive the influenza vaccine.

### Intent to receive the COVID-19 vaccine

**Figure 1:**
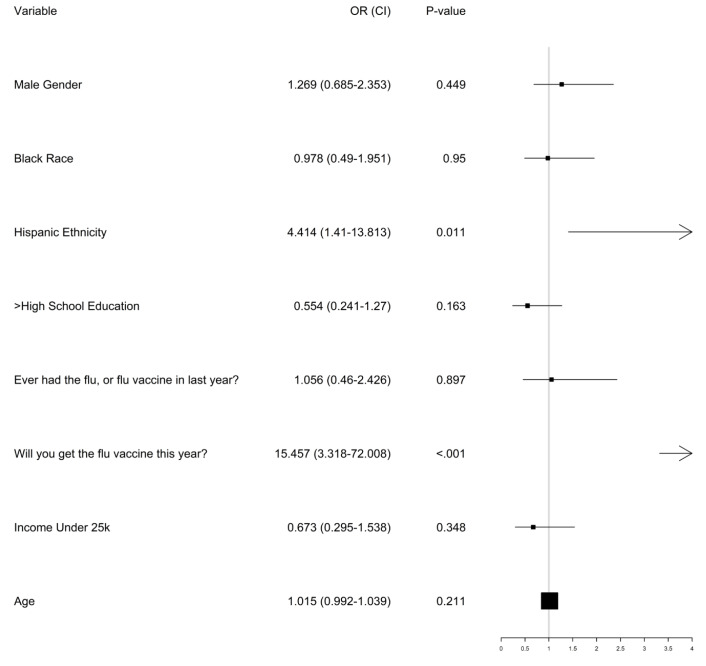
Multivariable logistic regression predicting COVID-19 vaccine interest.

Patients who reported intending to receive the vaccine against COVID-19 were significantly more likely to also report intention to receive the influenza flu vaccine in 2020 (*P* < 0.001). There was a statistical trend whereby patients who intended to receive the COVID-19 vaccine were less likely to be African American, more likely to be Hispanic, and have received the flu vaccine in the previous year. The results of the multivariable logistic regression to predict COVID-19 vaccine intent is reported in Figure 1.

*Intention to receive the influenza vaccine* was most associated with an increased likelihood of planning to receive the COVID-19 vaccine (odds ratio = 15.45, 95% CI = 3.3-72, *P* value<0.001). A total of 39% (77/197) of those who indicated they would take the flu vaccine would also take the COVID-19 vaccine compared to 4% (2/47) of those who did not have intent to take the flu vaccine. Patients with Hispanic ethnicity were also significantly more likely to say they would take an available COVID-19 vaccine (odds ratio = 4.14, 95% CI = 1.41-13.81, *P* value=0.011); 52% of the 23 Hispanic patients reported intent to take the vaccine, vs 31% in all others.

### Reasons for vaccine hesitancy

**Figure 2:**
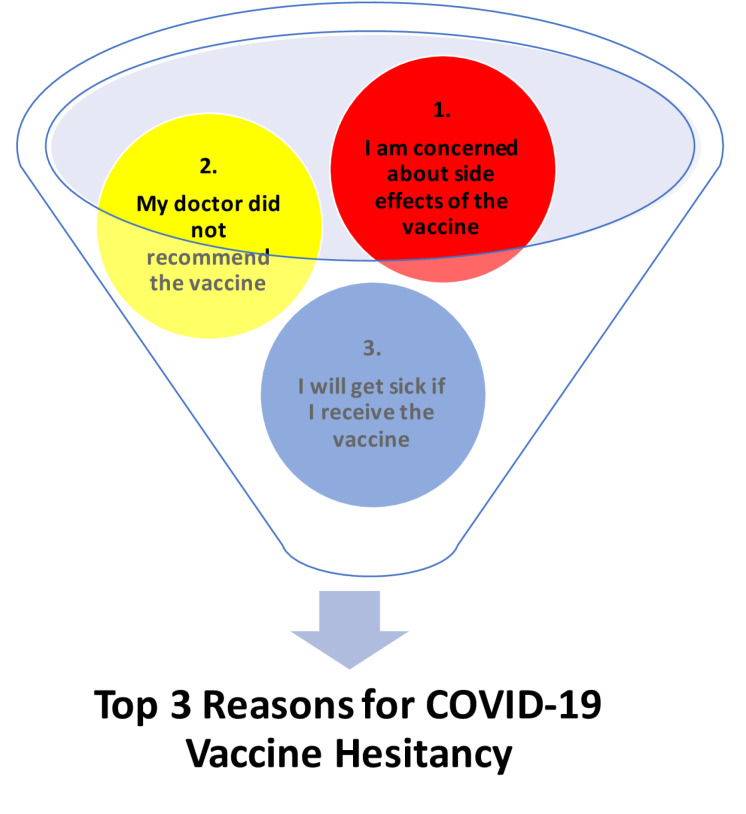
Reasons for COVID-19 vaccine hesitancy.

Of the 167 participants who were unsure or did not intend to receive the COVID-19 vaccination, 48% (n=80) attributed their hesitancy to concern with the side effects of the vaccine. The second most common reason for reporting no or unsure intention to be vaccinated against COVID-19 included fear of getting sick from the vaccine (n=25) and that their doctor did not recommend the vaccine (n=31) (Figure 2).

### Participants’ attitudes and beliefs regarding vaccines

Lastly, we investigated whether patients had different overall perceptions about vaccines, regardless of their interest in taking the COVID-19 vaccine (Table 2). For each question, we report the count (%) of patients who answered “agree” or “strongly agree”. Patients who intended to receive the COVID-19 vaccine answered “agree” or “strongly agree” at a significantly higher frequency for all questions except fear of flu vaccine side effects, fear of vaccine or COVID-19 vaccine side effects (significantly less).

Our findings underscore the significance of social factors in health. Previous researchers have documented lower rates of vaccination for other diseases (e.g. flu) in African Americans compared to Whites.^10^ Other studies have also noted that vaccine acceptance, particularly as it relates to COVID-19, is much lower in African American individuals.^11^ Though multifactorial, we suspect that vaccination hesitancy in African American patients is partly related to the historically unethical use of blacks in medical experimentation (i.e. Tuskegee experiment) and the subsequent distrust of the medical system in addition to systemic barriers limiting equal access to healthcare.^12,13^ Nonetheless, these disparities raise concern as the burden of COVID-19 morbidity and mortality has disproportionately affected African American populations. For example, in New York City, African Americans maintain the highest rates of COVID-19 related hospitalization and mortality compared to white persons.^12^ The cumulative effect of both increased vaccine hesitancy and increased disease burden has enormous potential to exacerbate existing health disparities occurring in black communities. As such, it is essential that vaccine administration strategies incorporate trusted community leaders, health workers and cornerstone resources in predominantly African American communities.

## Discussion

In the context of the current COVID-19 crisis, this study is among the first to assess intent to receive both the COVID-19 and influenza vaccines in adult clinic patients. Our survey sample is more racially diverse than previous studies, which is critical to address racial/ethnic disparities in COVID-19 prevalence, morbidity, and mortality.

Nearly two thirds (67%) of our participants indicated hesitancy to receive the COVID-19 vaccine, with only one third (33%) reporting intent to receive the COVID-19 vaccine. This finding is particularly alarming because our study was conducted from September through December 2020, when COVID-19 incidence and mortality rates in the United States were at an all-time high and at the dawn of vaccine distribution.^14^

U.S. studies evaluating the early attitudes of individuals towards a COVID-19 vaccine prior to vaccine distribution reported more optimistic findings compared to our present study. One email survey (n=316) reported that nearly 68% of participants indicated a positive likelihood of intending to be vaccinated against COVID-19.^15^ Similarly, a cross sectional survey (n=991) of U.S. participants taken in April of 2020 reported that over half (57.6%) intended to get vaccinated against COVID-19.^16^ The results of these studies may differ from our study because the majority of participants in these prior studies were white (63.27% and 63.3% respectively) and white communities have typically demonstrated greater vaccine uptake compared to other racial/ethnic groups. Further, these studies were conducted very early in the year when a COVID-19 vaccine was only hypothetical. As such, early findings regarding intent to vaccinate may not reflect current intent to vaccinate as the release of the vaccines for laypersons is now imminent.

Identifying the factors associated with increased vaccine hesitancy is critical to enable health professionals to create strategic approaches to vaccine education and promotion among patients, particularly racial/ethnic minority patients who bear a disproportionate burden of COVID-related morbidity and mortality. Our data reported a statistical trend of African Americans less likely to intend to receive the vaccine, suggesting that efforts to remove vaccine hesitancy needs to focus on African Americans.

The most common reason cited by participants who reported “no” or were “unsure” whether they would be vaccinated against COVID-19 included concerns about safety and the side effects of the COVID-19 vaccine, a result consistent with earlier vaccine studies.^17,18^ Increasing levels of transparency regarding vaccine safety may be an effective strategy to increase public vaccination acceptance of the COVID-19 vaccine.

The second most common reason for COVID-19 vaccine refusal was that the doctor did not recommend the vaccine, which reinforces the essential role of primary care physicians in promoting vaccination to patients. Preliminary studies have proposed that patients are more likely to receive a vaccine when recommended by a medical doctor compared to those whose doctor does not explicitly recommend vaccination.^19,20^ As the COVID-19 vaccine continues to be released to healthcare workers and will soon be available to laypersons, physicians will serve as the most important and trusted sources of vaccine information for laypersons.

The third most common reason for COVID-19 vaccine hesitancy included fear of getting sick from the vaccine. A substantial amount of research has shown that public concerns regarding the safety profile of vaccines and vaccine side effects are amongst most important variables that influence decisions to vaccinate, particularly for newly developed vaccines.^20-23^ For instance, in one telephone-based interview (n=1155) nearly 13% of participants reported intent to delay vaccination pending further verification of adverse events in others, while 17% reported no plans to receive vaccination.^24^ Acknowledging the above factors generated by laypersons, in addition to gauging both health literacy and vaccination literacy of laypersons is essential to adopting vaccine campaigns that are informative, effective and emphasize public reassurance in vaccine safety.

Our study has limitations. First, we used a convenience sample thus the results may not entirely reflect the general attitudes of the U.S. population. Further the sample size of our study may have been underpowered; yet significant results and statistical trends indicate that the sample size was adequate. Finally, our study measures assessed *intent* to receive a vaccine, which does not necessarily equate to the behavior of vaccine receipt.

A strength of our study is that the timing of the survey dissemination corresponded with a peak time of the pandemic in the United States. Additionally, we surveyed individuals about their intent to be vaccinated during a time when vaccines were emergently authorized and being prepared to distribute to frontline healthcare workers, making the findings particularly relevant. Further, the demographic composition of our survey group is more racially diverse than most U.S. communities. Given the disproportionate burden of COVID-19 on minority and underserved populations, our findings may be particularly may be useful in informing strategies to increasing vaccination in these target communities.

## Conclusion

In conclusion, we found that majority of participants (67%) in the survey conducted during the height of the COVID-19 pandemic would be hesitant to accept vaccination against COVID-19, while most (81%) still intend to receive the influenza vaccine. There was a statistical trend that blacks were less likely to intend to receive the vaccine, which is concerning given the disproportionate effects of COVID-19 and influenza on African America communities. Our results indicate that majority of participants who demonstrated some hesitancy (answering “no” or “unsure”) may be amenable to vaccination many if there is assurance of the vaccine safety profile, the vaccine is explicitly recommended by their medical doctor and there is greater education that the vaccine will not induce infection with the disease. While healthcare employees are currently receiving the first wave of the COVID-19 vaccine, simultaneous efforts are urgently needed to create strong messages that will facilitate decreased vaccine hesitancy and improve vaccine acceptance among U.S. laypersons.

### Key recommendations

- Urge partnership of researchers and local health workers to coordinate culturally appropriate community vaccination education/promotion programs.
- Create and disseminate vaccine educational material with an emphasis on incorporating plain language and easy to read content.
- Authorize use of community cornerstones to serve as sites of vaccine administration and education.
- Provide simple fact sheets to local citizens demystifying common myths regarding the COVID-19 and influenza vaccines.
- Ensure that vaccines are equally accessible and affordable for laypersons.
- Empower primary care physicians with essential tools to provide vaccine education and promote vaccine administration.

## Acknowledgements

The authors express gratitude to the study participants who took time to complete the questionnaire. Further, we would like to thank Joy Stacker, Tonette Chapman, Yessica Alvarez, Armoni Butler, Isrelis Navarro, Cintreen Harris and the LSU Medicine clinic staff for their hard work and support of the study.

## Funding

None.

## Competing interests

The authors declare that there is no conflict of interest.

## Ethical approval

Herein, we reports research that included human participants and data. The study (protocol number: 1348) was determined to be exempt by the Louisiana State University Health Sciences Center- New Orleans Institutional Review Board and has determined to be exempt. Informed consent was obtained from study participants prior to data collection.

We adhered to all ethical standards enforces by our review committee.

## Authors’ contributions

KJ, AC, AM and KY conceived and designed the study. KJ, JA, OA and CW conducted the literature search, and collected and organized data. AC, KY assisted in the data analysis, statistical analysis and data interpretation. CW, OA, JH, KJ prepared tables and figures. KJ, OA, AM, JH, JA, KY and CW assisted in manuscript preparation and devised the initial version of the manuscript. KJ, AM, KY, CW, JH, OA, JA and AC edited, revised and approved the final draft of the article.

**Table 1:** Patient Characteristics by Intent to Receive the COVID vaccine

**Variable**	**All (248)**	**Yes (81)**	**No/Unsure (167)**	***P*** ** value**
Male gender	104 (42.3, 2)	36 (45, 1)	68 (41, 1)	0.583
Female/other gender	142 (57.7, 2)	44 (55, 1)	98 (59, 1)	
Race				0.064
Black	161 (65.2, 1)	46 (56.8)	115 (69.3, 1)	
White	59 (23.9, 1)	22 (27.2)	37 (22.3, 1)	
Asian	14 (5.6)	6 (7.4)	8 (4.8)	
Other	13 (5.2)	7 (8.6)	6 (3.6)	
Hispanic ethnicity	23 (9.4, 3)	12 (14.8)	11 (6.7, 3)	0.06
Education level				0.3779
No education	34 (13.8, 2)	15 (18.8, 1)	19 (11.4, 1)	
High school	123 (50, 2)	39 (48.8, 1)	84 (50.6, 1)	
Some college	72 (29.3, 2)	21 (26.2, 1)	51 (30.7, 1)	
College/professional graduate	17 (6.9)	5 (6.2)	12 (7.2)	
Will you get the Flu Vaccine?	197 (80.7, 4)	77 (97.5, 2)	120 (72.7, 2)	<0.001
Have you received flu shot in last year?	174 (70.4, 1)	64 (79)	110 (66.3, 1)	0.053
Ever had Flu?	42 (16.9)	10 (12.3)	32 (19.2)	0.209
Income Under 25k	159 (66, 7)	50 (64.1, 3)	109 (66.9, 4)	0.666
Age (y)				
18-29	10 (4.1, 4)	1 (1.2)	9 (5.5, 4)	0.205
30-44	49 (20.1, 4)	16 (19.8)	33 (20.2, 4)	
45-60	84 (34.4, 4)	27 (33.3)	57 (35, 4)	
>60	101 (41.4, 4)	37 (45.7)	64 (39.3, 4)	

**Table 2:** Attitudes and beliefs towards vaccines, COVID-19 and influenza

**Question**	**All (248)**	**Yes (81)**	**No/Unsure (167)**	***P*** ** value**
All vaccines offered by my health care providers are beneficial	158 (63.7)	73 (90.1)	85 (50.9)	<0.001
I do what my health care provider recommends about vaccines	177 (71.4)	72 (88.9)	105 (62.9)	<0.001
I am concerned about serious side effects of vaccines	146 (58.9)	28 (34.6)	118 (70.7)	<0.001
Getting vaccines is a good way to protect myself from disease	170 (68.5)	73 (90.1)	97 (58.1)	<0.001
The information I receive about vaccines. From my health care provider is reliable and trustworthy	171 (69)	74 (91.4)	97 (58.1)	<0.001
Getting myself vaccinated is important for the health of others in the community	179 (72.2)	75 (92.6)	104 (62.3)	<0.001
Vaccines are effective	168 (67.7)	75 (92.6)	93 (55.7)	<0.001
Vaccines are important for my health	173 (69.8)	76 (93.8)	97 (58.1)	<0.001
The COVID-19 vaccine offered by my healthcare provider is beneficial	93 (37.5)	72 (88.9)	21 (12.6)	<0.001
I do what my healthcare provider recommends about the COVID-19 vaccine	105 (42.3)	73 (90.1)	32 (19.2)	<0.001
I am concerned about serious side effects of the COVID-19 vaccine	154 (62.1)	22 (27.2)	132 (79)	<0.001
Getting the COVID-19 vaccine is a good way to protect myself from disease	97 (39.1)	73 (90.1)	24 (14.4)	<0.001
The information I receive about the COVID-19 vaccine from my healthcare provider is reliable and trustworthy	95 (38.3)	74 (91.4)	21 (12.6)	<0.001
Getting myself vaccinated for COVID19 is important for the health of others in the community	100 (40.3)	76 (93.8)	24 (14.4)	<0.001
The COVID-19 vaccine is effective	84 (33.9)	71 (87.7)	13 (7.8)	<0.001
The COVID-19 vaccine is important for my health	92 (37.1)	73 (90.1)	19 (11.4)	<0.001
The flu vaccine offered by my healthcare provider is beneficial	169 (68.1)	75 (92.6)	94 (56.3)	<0.001
I do what my health care provider recommends about the flu vaccine	168 (67.7)	74 (91.4)	94 (56.3)	<0.001
I am concerned about serious side effects of flu vaccine	104 (41.9)	28 (34.6)	76 (45.5)	0.131
Getting the flu vaccine is a good way to protect myself from disease	167 (67.3)	74 (91.4)	93 (55.7)	<0.001
The information I receive about the flu vaccine from my health care provider is reliable and trustworthy	174 (70.2)	75 (92.6)	99 (59.3)	<0.001
Getting myself vaccinated for flu is important for the health of others in the community	173 (69.8)	77 (95.1)	96 (57.5)	<0.001
The flu vaccine is effective	166 (66.9)	75 (92.6)	91 (54.5)	<0.001
The flu vaccine is important for my health	169 (68.1)	77 (95.1)	92 (55.1)	<0.001
